# Breast cancer detection by analyzing the volatile organic compound (VOC) signature in human urine

**DOI:** 10.1038/s41598-022-17795-8

**Published:** 2022-09-01

**Authors:** Judit Giró Benet, Minjun Seo, Michelle Khine, Josep Gumà Padró, Antonio Pardo Martnez, Fadi Kurdahi

**Affiliations:** 1 Center for Embedded Cyber-Physical Systems (CEPS), University of California Irvine (UCI), Irvine, 92697 USA; 2 Department of Biomedical Engineering, University of California Irvine (UCI), Irvine, 92697 USA; 3 South Catalonia Oncology Institute (IOCS), Sant Joan de Reus University Hospital, IISPV, Rovira i Virgili University, 43204 Reus, Spain; 4 Department of Electronic and Biomedical Engineering, Universitat de Barcelona (UB), 08028 Barcelona, Spain

**Keywords:** Biochemistry, Cancer, Biomarkers, Health care, Medical research, Oncology, Chemistry, Energy science and technology, Engineering, Mathematics and computing

## Abstract

A rising number of authors are drawing evidence on the diagnostic capacity of specific volatile organic compounds (VOCs) resulting from some body fluids. While cancer incidence in society is on the rise, it becomes clear that the analysis of these VOCs can yield new strategies to mitigate advanced cancer incidence rates. This paper presents the methodology implemented to test whether a device consisting of an electronic nose inspired by a dog’s olfactory system and olfactory neurons is significantly informative to detect breast cancer (BC). To test this device, 90 human urine samples were collected from control subjects and BC patients at a hospital. To test this system, an artificial intelligence-based classification algorithm was developed. The algorithm was firstly trained and tested with data resulting from gas chromatography-mass spectrometry (GC–MS) urine readings, leading to a classification rate of 92.31%, sensitivity of 100.00%, and specificity of 85.71% (N = 90). Secondly, the same algorithm was trained and tested with data obtained with our eNose prototype hardware, and class prediction was achieved with a classification rate of 75%, sensitivity of 100%, and specificity of 50%.

## Introduction

The US female population is 165.92 million women. The US Preventive Services Task Force (USPSTF) recommends biennial screening mammography for women aged 50–74 and recommends against screening younger and older women due to current evidence being insufficient to prove its benefits^1^. This reduces BC mortality by 15%^2^. According to the CDC, only 66% of women over 40 years of age do attend BC screenings annually or biannually^3^. The main reason why women skip their mammogram appointment is pain^4^. In the US, 451,936 BCs are detected yearly^5^, 150,000 of which are detected late-stage because of mammogram absenteeism or inefficiency of current methods. In fact, the impact of implementing a mammogram-based screening on breast cancer mortality has been under discussion for a very long time. A review by Moss et al. suggests that although most statistical studies report a decrease in breast cancer mortality over the years, such a decrease can also be observed before the implementation of screening and in age ranges excluded from screening^6^.

If BC is detected in an early stage, its metastasis risk is minimal as well as the likelihood of a subsequent metastasis, patient suffering and death. If detected in a later stage, however, patients will require treatment and potentially a mastectomy, which has a notorious impact on women’s mental health. These interventions have an approximate cost of $25,000 per patient. Considering that 80% of the US population is covered by insurance, health insurances could save over $3 billion yearly if all BCs detected in their patients were detected in an early stage. Additionally, apart from the mastectomy, if cancer progresses and the patient lives with metastasis, the cost of her treatment (monoclonal antibodies, sometimes conjugated with chemotherapy, cyclines inhibitors, hormone therapy, palliative radiotherapy...) is notorious. Blumen et al. report yearly costs per patient of up to $82,121 for BCs detected in stage I/II as opposed to $129,387–$134,682 if detected in stage III/IV, mainly due to chemotherapy^7^.

BC is the leading cause of death by cancer in women under 40 years of age, especially among African–American women. Young women present a denser mammary tissue than post-menopause women, thus decreasing mammogram’s sensitivity and specificity in small tumors^8^. In addition to that, their tissue is more sensitive to mammography’s radiation dose^9^. Although its dose is not substantial enough to be considered harmful, biennially exposure to mammography could increase BC risk^10,11^. This observation, shared by many authors, started a debate on the worthiness of mammogram-based BC screenings^12^. Furthermore, in 2017, the World Health Organization (WHO) published the “WHO Position paper on mammography screening”. Reference^13,14^ stating an urgent need for a new radiation-free and sensitive BC screening solution. In other words, although mammography is currently the best solution to reduce BC mortality, there is still room for improvement.

### Current methods for breast cancer screening

Most healthcare systems base their BC screening programs on image-based technologies, i.e. mammography, ultrasonography and magnetic resonance imaging (MRI). The most common reason why women seek medical advice related to BC is having detected a mass in their breasts. However, 90% of these will be found to not be cancer but other benign lesions such as fibroadenoma^15^. Actually, image-based techniques typically fail to correctly identify tumors in women with fibrocystic breasts (breast tissue with healthy lumps) because of highly dense breast tissue^16^. Around 53–60% of women worldwide have this condition^17^. This fact does not only lead to numerous false positives but also increases the recommended screening frequency (and thus the potentially cancerogenic small radiation dose).

When the tumor is small or the mammary tissue is dense, MRI is typically the preferred approach, since it is highly specific and sensitive in addition to being a radiation-free technique. For this reason, BRCA1 or BRCA2-positive women (who present a high risk of developing BC) are screened from a younger age using MRI. Women with fibrocystic mastopathy (whose breasts have difficult tumor visualization) and patients whose mammogram results are inconclusive are typically screened with MRI as well^18^. However, screening 1 M women costs $640 M and $216 M if done with MRI and mammography respectively^19^. There is therefore a tendency of enhancing mammography by processing the resulting image with an AI-based classification algorithm. However, even if this solution can potentially greatly increase the mammogram’s sensitivity, it does not solve another problem: this technique is painful to some women and thus causes screening absenteeism.

### State of the art: electronic noses

Even though there do exist some publications on odor-based cancer screenings—i.e. electronic noses or eNoses, the tech transference rate is extremely low. The first eNose, proposed by Persaud and Dodd^20^ in Nature in 1982, was designed to loosely mimic the human olfactory pathway. An eNose is based on a sensor array that responds differently according to specific VOCs. In 2014, Asimakopoulos et al.^21^ set the race towards an eNose approach for cancer screening with the first study on an eNose-based prostate cancer identification, achieving a specificity of 93% (n = 41). Furthermore, Guerrero-Flores et al.^22^ describes a cervix cancer screening in which a dog detects cancer cells in blood by smelling it. Buszewski et al.^23^ also presents a VOC-based screening from breath through canine smell and Blatt et al.^24^ describes the same screening carried out by an eNose. Phillips et al.^25^, describe a BC screening using an electronic device that analyzes the patient’s breath. Burton et al.^26^ and Guo et al.^27^ among others report on some biomolecules that could allow a BC screening from urine samples. In this case, not only does it detect the presence of cancer but also the stage: I and II (early) versus III and IV (advanced). However, in these studies, the dogs/eNoses may have responded to odors associated with cancer, such as inflammation or metabolic products, rather than specifically to cancer itself^28^. Hence, the future of volatilome-based screening should not seek a cancer odor but rather a specific VOC pattern for each cancer type, which has already been proven feasible with BC among others^29^.

### Technologies for artificial odoring

**Figure 1:**
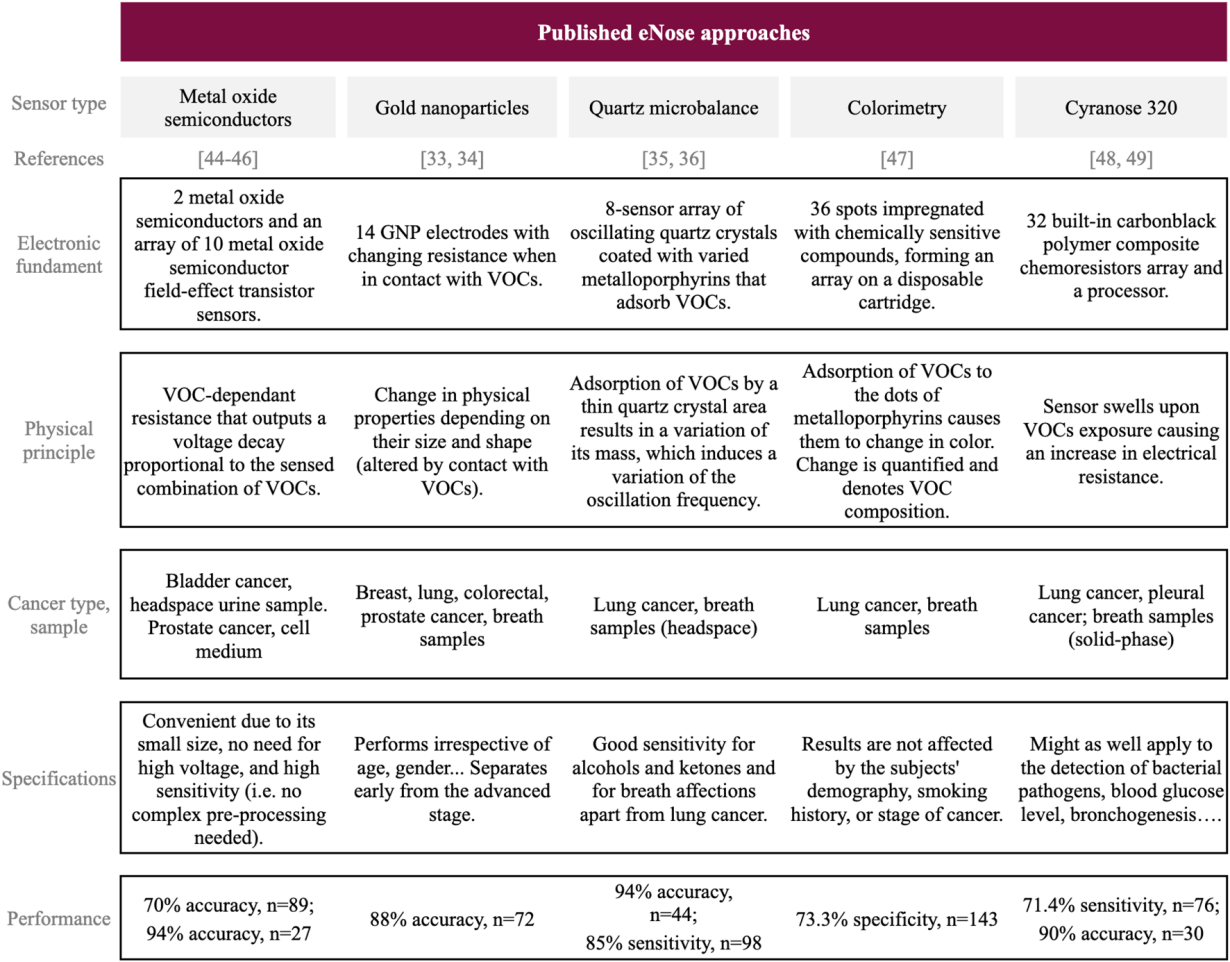
Summary of published eNose approaches to portable low-cost screening solutions: Metal oxide semiconductors^43–45^, gold nanoparticles^34,35^, quartz microbalance^36,37^, colorimetry^46^ and the *Cyranose 320*^47,48^.

Some electronic noses have already reached the industry: vReCIVAő Breath Sampler by Owlstone Medical analyzes breath to detect lung cancer. Aeonose is a certified medical device that can screen colorectal cancer with a sensitivity of 95% and specificity of 64% and advanced adenomas with sensitivity and specificity of 79% and 59% respectively (N = 511) from exhaled-breath^30^. NASA has also developed an eNose, a smartphone-based device to monitor air quality inside spacecraft. And they have recently modified it to screen people for COVID-19 in a low-cost and efficient manner. Heracles by Alpha MOS is an electronic nose based on ultra-fast gas chromatography that classifies wines^31^, olive oil^32^ and other substances. Although being the best-performing approach according to most authors^33^, metal-oxide sensors are not the only low-cost and portable solution for artificial odoring. An eNose based on a nanosensor array with gold nanoparticles (GNP)^34,35^ or a quartz microbalance^36,37^ are different types of sensors that have proven high sensitivity and specificity applied to BC and lung diseases respectively. Peng et al.^34^ present a tailor-made array of cross-reactive nanosensors based on organically functionalized gold nanoparticles and the GC–MC technique (GC–MS) that distinguishes the breath patterns of different cancers. Figure 1 summarises various eNose descriptions found in bibliography.

### Aims of our study

The medical community has long accepted the fact that BC produces metabolic changes in human physiology^27–41^. More specifically, Vignoli et al. emphasise on a ”BC metabolomic signature in breast tissue, blood, serum/plasma and urine”, detected through Nuclear Magnetic Resonance spectroscopy (NMR)^42^. A metanalysis by Dent et al.^33^ reviews the findings from several authors from 2003 up to 2012 and concludes that the ”VOC fingerprint” differs significantly among publications. In conclusion, the key to successfully identifying cancer is focusing on the proportion between VOCs rather than on VOCs themselves.

The aim of this study is therefore to obtain multidimensional data related to urine smell and analyze it using statistical algorithms. Like the human nose, the implemented software will respond in concert to a given set of odors—a pattern or *smellprint*—which will be analyzed, compared with stored patterns, and recognized. The device under study is based on the principle that BC causes certain inflammatory processes, resulting in specific circulating metabolites. These subsequently interact with the excretory system, which translates into the urine of BC patients containing the decomposition products of these metabolites.

## Results

### Software considerations: classic biostatistics approach

**Figure 2:**
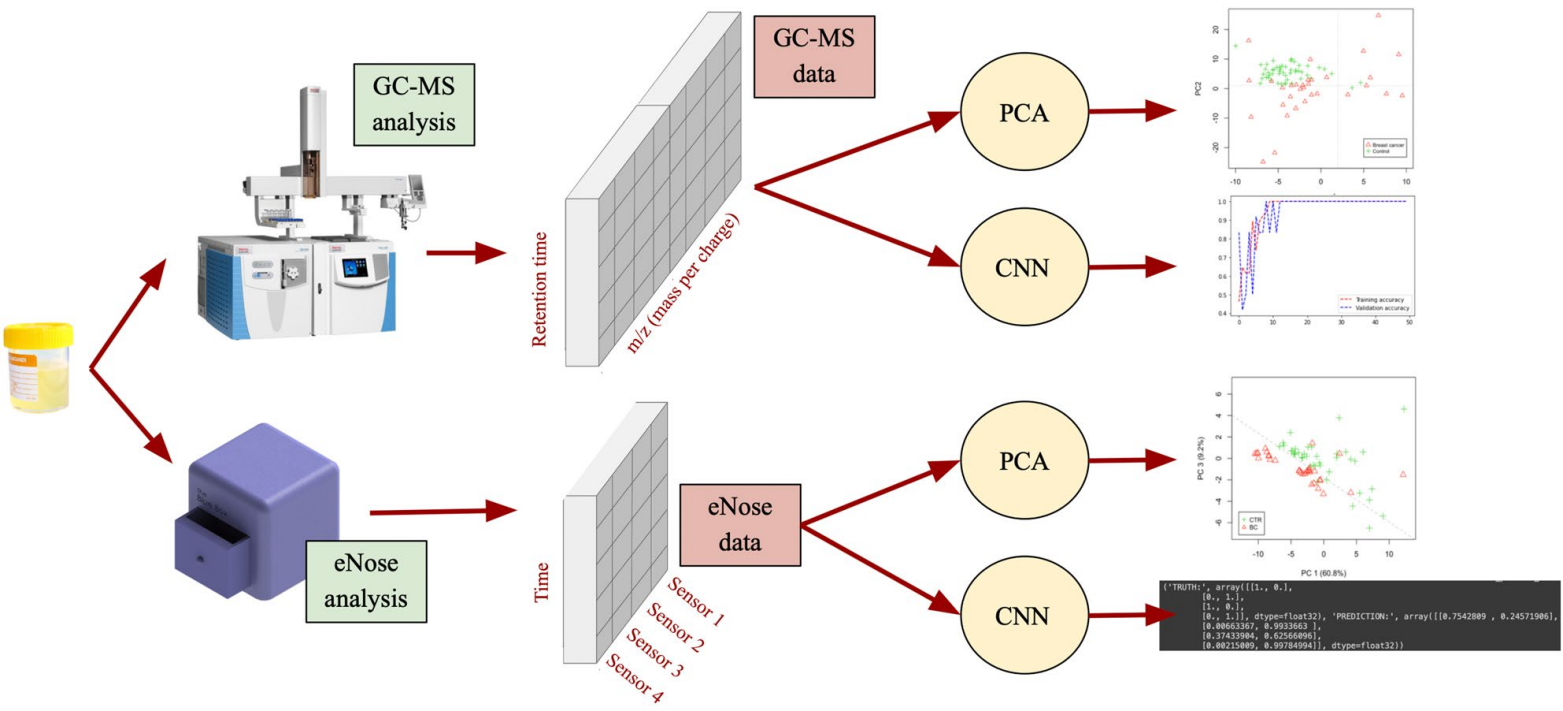
Design of the project. Upon collection, urine samples were analyzed using both an eNose and a GC–MS. Data resulting from the GC–MS analysis contain information relative to the m/z of the VOCs found in the sample per unit of time (retention time). The output of the eNose analysis was a 2D array that was informative of the intensity of each sensors’ reaction as a function of time. Finally, both datasets were input to a biostatistics-based classification (PCA) as well as an AI-based classification (CNN).

**Figure 3:**
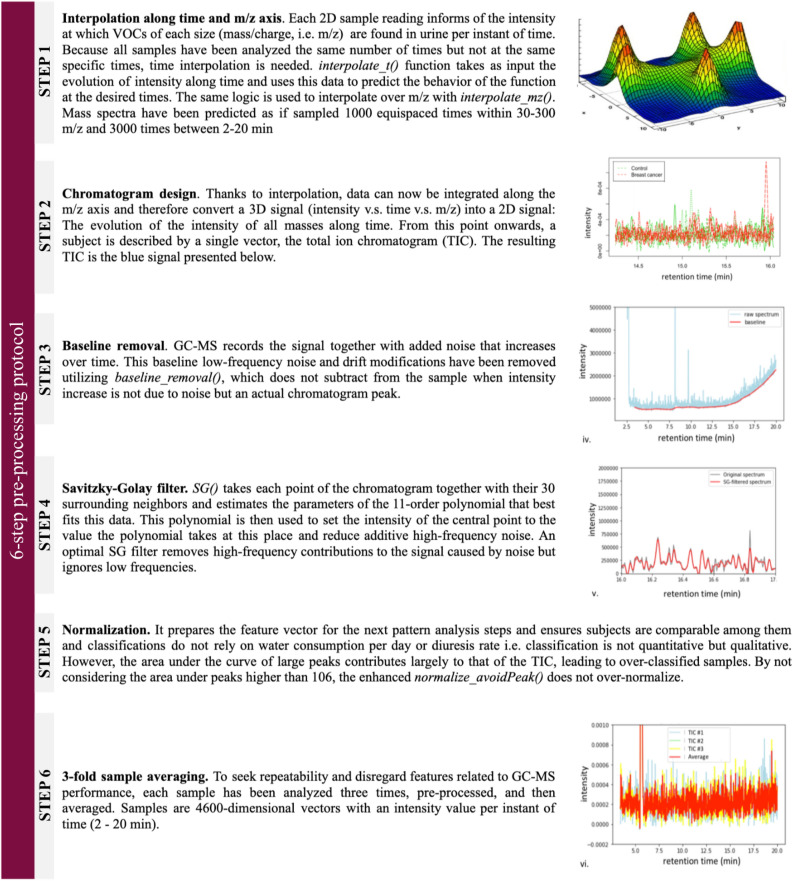
Summary of the pre-processing steps from GC–MS raw data reading up to total ion chromatogram (TIC) design. Sample 151 (belonging to a breast cancer subject) has been used to illustrate those steps.

As depicted in Fig. 2, the first approach consisted of building a first model for sample classification based on classic biostatistics. Before classifying the data, it was pre-processed to ensure that the classification would, later on, rely on the characteristic of cancer instead of on irrelevant artifacts. Pre-processing steps are described in Fig. 3. Later on, a Principal Components Analysis (PCA) was performed, and it was used to prove the hypothesis mentioned below:

- Urine samples from breast cancer patients are significantly different from control samples.

**Figure 4:**
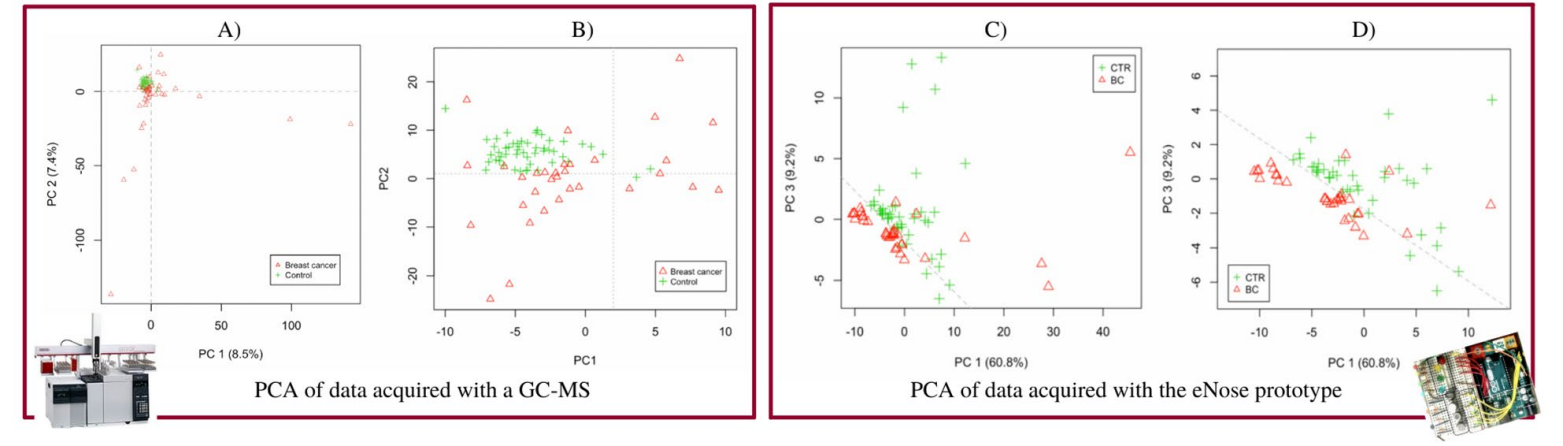
(**A**) PCA performed on data obtained by analyzing control (green) and BC (red) human urine with a GC–MS. (**B**) Figure on the right is a zoom of the figure on the left. (**C**) PCA performed on data obtained by analyzing control (green) and BC (red) human urine with the eNose prototype. (**D**) Figure on the right is a zoom of the figure on the left.

The PCA was firstly conducted using GC–MS data. A visual inspection of this classification is displayed in Fig. 4A,B. Once observed that it was possible to discriminate control samples from BC samples, the same procedure was repeated by analyzing the same data with the eNose prototype. This time, the classification algorithm was simplified so that it could run on the edge—e.g., in the microcontroller inside the eNose. It was thus a simplified PCA, consisting of only 2 Principal Components (PCs). The projection of urine samples against PC1 and PC2 is shown in Fig. 4C,D.

Hence, the scientific principle for BC eNose was proved. Below we compare the *smellprint*, obtained from two data sources: GC–MS-acquired data (Fig. 4A,B), versus the eNose-captured signal (Fig. 4C,D).

### Software considerations: a machine learning approach

**Figure 5:**
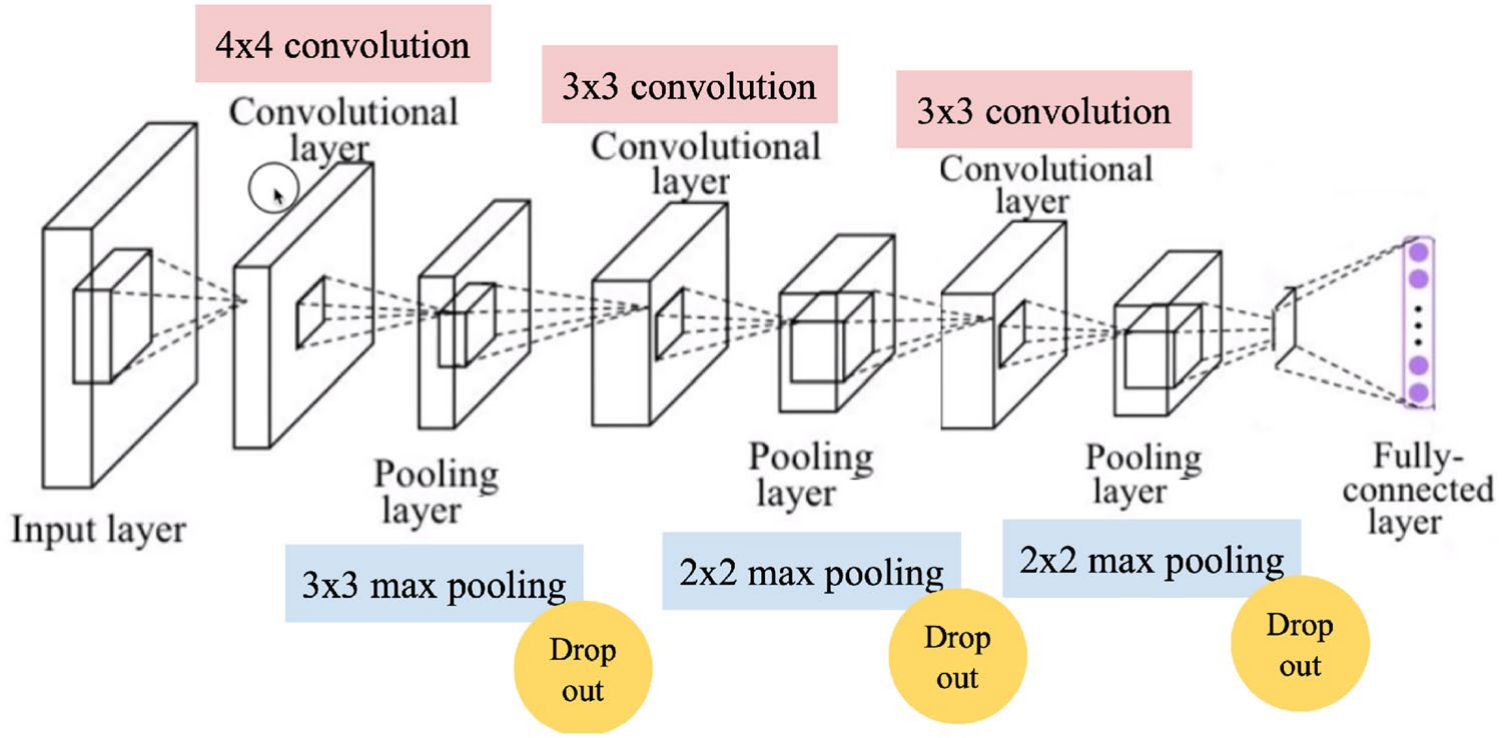
Convolutional neural network used for sample classification, consisting of convolutional filters of size 32, 64, 128 and linear activation. The output layer of the neural network presents two cells (two outputs) with a softmax activation function.

Once the null hypothesis has been tested, an AI-based algorithm has been implemented to assert the classification capacity of a convolutional neural network (from now on, CNN). This neural network consisted of four convolutional filters of size 32, 64, 128 and linear activation. The output layer of the neural network presented two cells (two outputs) with a softmax activation function. Dropout was implemented to avoid model overfitting. Figure 5 is a sketch of how the model structure was designed. In our first approach to the CNN (ConvNet) model, GC–MS data was used: 90 urine samples from control and BC subjects. This model was trained with 50 epochs of batch size 10. Out of 90 samples, 65 (72.3%) samples were used for training, 12 (13.3%) for testing, and 13 (14.4%) for cross-validation. The achieved training accuracy was 98.20%, and the training loss was 7.70%. As far as validation is concerned, the output of the model (with GC–MS data) was 6 true positives, 6 true negatives, 0 false negatives and 1 false positive, presented in a confusion matrix in Table 1. Hence, the *validation accuracy was 92.31%*. As displayed in Table 3, the model has a *sensitivity of 100% and a specificity of 85.71%*.

**Table 1:** Confusion matrix of the CNN model using GC–MS data.

	(+) predicted	(−) predicted
(+) ground truth	6	0
(−) ground truth	1	6

Out of 90 samples, 65 (72.3%) samples were used for training, 12 (13.3%) for testing and 13 (14.4%) for validation.

Similar to the previous section, the model was subsequently trained and tested with eNose-obtained data. Out of 44 samples, 36 (81.8%) samples were used for training, 4 (9.1%) for testing, and 4 (9.1%) for validation. The structure of the model was the same as presented in Fig. 5. The achieved training accuracy was 93.3% and the training loss was 14.72%. As far as validation is concerned, the output of this model consisted on 2 true positives, 1 true negative, 1 false positive and 0 false negatives. These results are presented in a confusion matrix in Table 2. Validation accuracy was 75.00%. As displayed in Table 3, the model has a sensitivity of 100.00% and specificity of 50.00%.

**Table 2:** Confusion matrix of the CNN model using data acquired by the eNose prototype.

	(+) predicted	(−) predicted
(+) ground truth	2	0
(−) ground truth	1	1

36 (81.8%) samples were used for training, 4 (9.1%) for testing and 4 (9.1%) for validation.

### Accuracy, sensitivity and specificity

Upon collection, urine samples were first analyzed using a gas chromatographer–mass spectrometer (GC–MS), which provided an insight into the intensity in which every type of molecule was found at every retention time. In other words, the output consisted of a report for every retention time, which indicated the number of molecules found at that given time for every molecular mass, relative to their ionized charge. Figure 2 shows the shape of the GC–MS data. Later on, this data was processed by a PCA and CNN, which will be detailed as follows. Secondly, urine samples were also analyzed utilizing the eNose prototype that we developed. Please refer to “Methods” section for a detailed description of the prototype. The output of this second analysis was a 2D matrix containing the response of the 4 sensors at every instant of time. As one can observe in Fig. 2, this data was classified by a PCA and a CNN as well. Table 3 displays the accuracy of the two models that were described before: the biostatistics model and the AI-based model respectively.

**Table 3:** Sample classification using biostatistics (PCA) and machine learning (cNN) vs reported performance of mammography.

	Technique	Accuracy (%)	Sensitivity (%)	Specificity (%)	Sample size
PCA	GC–MS data	77.11	75.05	68.33	N = 90
	eNose data	58.30	75.00	45.00	N = 44
cNN	GC–MS data	92.31	100.00	85.71	N = 90
	eNose data	75.00	100.00	50.00	N = 44
Mammography	–	> 91.00^8^	86.90^42^	74.00–98.00^8^	–

As one can observe, the introduction of neural networks plays a critical role in enhancing the classification capability of the system. Additionally, it should also be noted that the model classification rate is highly dependent on the sample space size. Therefore we consider it necessary to conduct further studies to keep training the algorithm and achieve a sensitivity and specificity that are acceptable for an oncology screening.

## Discussion

As these results denote, machine learning—in this case, CNN—causes a significant impact on sample classification: In the presented case, the accuracy of the model rises from 58.30 to 75.00% (when using eNose data). Additionally, it should be noted that the eNose data set has a smaller dimensionality. Further, because this technology is intended for screening, special attention should be placed on sensitivity rather than specificity. In our case, if we compare our AI-powered model to mammography, our sensitivity is notably higher (100%). Mammography has a sensitivity of 86.9% and its performance is highly dependent on age and tissue density^42^. On the other hand, our highest specificity value is achieved when implementing CNN with GC–MS data.

In conclusion, the experiments carried out in this paper indicate that the implementation of AI in the medical field can yield new approaches and discoveries that classic biostatistics could not reach. This paper also suggests the feasibility of a future potential medical device for in-home and non-irradiating BC screening. Additional studies are yet to be conducted to better identify the classification capacity of the technology. The results presented above were achieved with a sample size of 90 patients. The authors believe that if the study was continued and a bigger sample size was achieved (of a magnitude of 300–500 patients), better classificationresults might be obtained.

In addition to the latter, the authors also conclude that there is also room for improvement considering both hardware and sensorics of the presented technology: The current technology solely uses commercially-available sensors, which are sensitive to a wide range of VOCs (very sensitive but poorly specific). As a result, future steps to improve the detectability of our system (e.g., building new sensors specifically designed for VOCs that are breast cancer biomarkers) might result in a better classification rate. In fact, the sensor’s response is not unequivocally correlated to the concentration of a single VOC but rather a *smellprint* consisting of a wide range of volatile BC biomarkers.

The study of urine smellprint and its correlation to breast cancer has been under study for a long time. However, a variety of electronic noses described in bibliography are subject to urine or exhaled alveolar breath and commonly apply to a wide variety of diseases, but none of them are designed specifically for BC. Urine metabolome is highly dependent on diet, environment and lifestyle, and consequently has more daily variability than serum or plasma^42^. It is for this reason that the metabolic fingerprint of BC in urine has been less studied than that of serum, plasma and breast tissue, but Vignoli et al. highlight the potential of this becoming a new tool for early-stage BC screening and report the findings from 4 different studies that coincide on the following: BC is associated with downregulation of acetate, alanine, creatinine, dimethylamine, glutamine, glycine, guanidoacetate, hippurate, isoleucine, lactate, leucine, succinate, taurine, threonine, trimethylamine n-oxide and valine^42^. The authors therefore believe that further research on urine characterization is yet another opportunity for improvement. Relevant knowledge related to urine characterization to date is presented as follows.

### 8-oxodG as a breast cancer volatile biomarker

**Figure 6:**
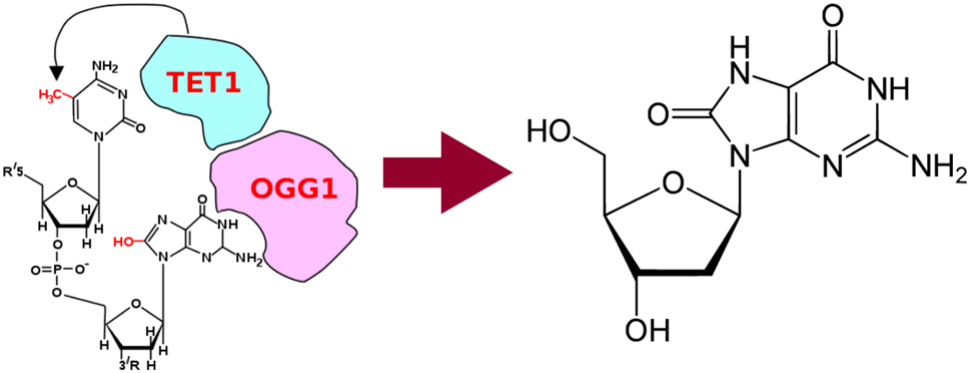
Formation of 8-oxodG out of guanine from a DNA sequence with intervention of TET1 and OGG1. 8-oxodG is a sensitive marker of DNA damage due to hydroxyl radical attack at the C8 of guanine. This damage is mutagenic and promotes cancer.

When it comes to BC, publications on its specific biomarkers are currently rare yet consistent. Some articles point out that an increased presence in urine of the volatile 8-oxo-7,8-dihydro-2’-deoxyguanosine, shown in Fig. 6, (from now on, 8-oxodG) denotes cancer. Guo et al.^27^ found a significant increase of 8-oxodG in patients with early-stage BC (p < 0.001) by ultraperformance liquid chromatography-electrospray ionization tandem mass spectrometry combined with a solid-phase microextraction (n = 184). According to another publication by Guo et al., reactive oxygen species (ROS) are produced by endogenous oxygen metabolism, as well as after exposure to ionizing radiation and chemical carcinogens^49^. The enzyme TET1 catalyzes a reaction with a guanine. For this reaction to happen, the guanine needs to have been oxidized to 8-hydroxy-2′-deoxyguanosine (8-OHdG or its tautomer 8-oxodG) due to the presence of a ROS. This oxidation allows enzyme OGG1 to bind to 8-oxodG and thus recruits TET1, which oxidizes the molecule, see Fig. 6 Increased ROS can cause oxidative base modifications and thus lesions in DNA. Since guanine exhibits the lowest oxidation potential, it is more vulnerable to free a radical, leading to the formation of 8-oxodG, commonly conceived as a biomarker of oxidative damage to DNA and a mutagen that contributes to carcinogenesis. Those features imply that a urine-based screening for 8-oxodG would be non-invasive, the patient would not get irradiated and it would not result in false positives, with the consequent economic savings^22,27,50^.

Although being one of the most reliable BC biomarkers, 8-oxodG has a significant size (average molecular weight of 283.2407), and is therefore poorly volatile. For this reason, the authors suspect that when samples were heated up, its concentration in urine headspace was very low. This is an additional opportunity for improvement: designing a system that can heat the sample more, or designing a sensor that is more sensitive to this molecule.

### Benzoic acid absence as a breast cancer volatile biomarker

Benzaldehyde is the only downregulated component by BC. It is absorbed via the gastrointestinal tract, skin and lungs, then distributed—especially in the blood and kidneys, and finally excreted very rapidly almost exclusively via urine. During the process, benzaldehyde gets oxidized to benzoic acid^51^. Hence, if Lavra et al.^29^ reported a lack of benzaldehyde in BC cells mediums, a lack of benzoic acid should be expected in BC patients’ urine. In fact, the PubChem database^52^ confirms benzoic acid presence in control urine within the range 350–630 nmol/mmol creatinine. Therefore, some of the sensors inside the analysis chamber have been specifically selected to react to benzenes and benzoic acid.

### Theoretical fundament for 2-nonanone presence in breast cancer urine samples

Lavra et al.^29^ suggests 2-nonanone, 4-methil-2-heptanone, isobutyric acid allyl ester, 1,3-dis-ter-butylbenzene and benzaldehyde among others as cancer biomarkers encountered directly in BC cell mediums. As depicted in Table 4, such components exhibit a significant difference in concentration between control cell medium and primary tumor cell medium (BT474) with respective p-values of 6.5.10$^{-21}$, 5.8.10$^{-11}$, 3.6.10$^{-10}$, 1.8.10$^{-9}$ and 0.013. Table 1 shows a summary of the different VOCs found in human physiology according to many authors.

**Table 4:** Intensity of concentration of various BC biomarkers encountered in cell media: control, benign breast tissue (MCF10A), and cancerous breast tissue (BT474).

	Bening breast tissue (MCF10A)	Cancerous breast tissue (BT474)	p-value
2-nonanone	Very low	Very high	6.5.10$^{-21}$
4-methil-2-heptanone	Very low	Very high	5.8.10$^{-11}$
Isobutyric acid allyl ester	Very low	Very high	3.6.10$^{-10}$
1,3-dis-ter-butylbenzene	Very low	Very high	1.8.10$^{-9}$
Benzaldehyde	Very high	Very low	0.013

### Acetone and 2-butanone as potential confounders in control urine

**Figure 7:**
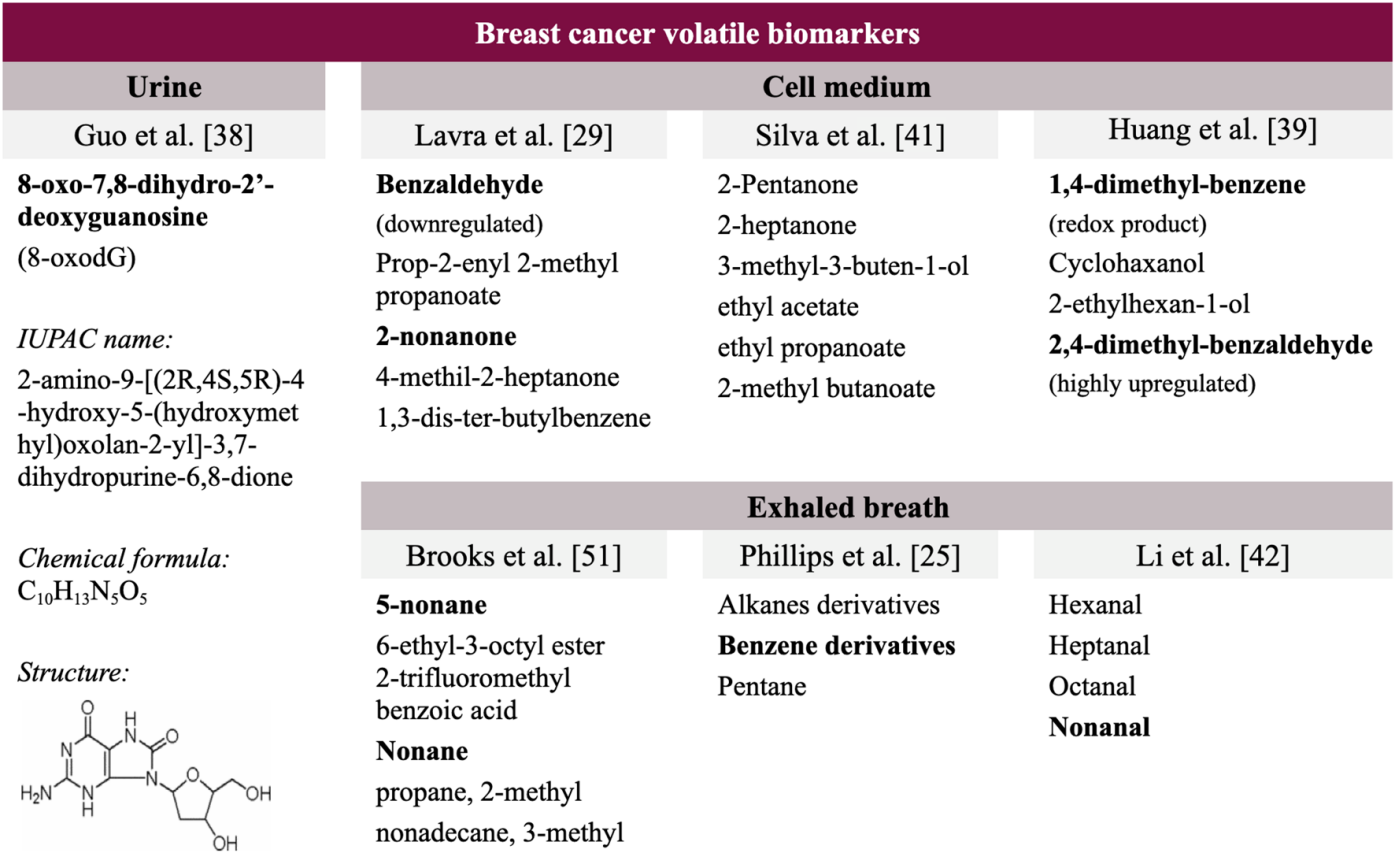
Summary of some VOCs encountered in urine, cell medium and expired breath, according to Guo et al.^27^, Lavra et al.^29^, Silva et al.^40^, Huang *et al*.^38^, Brooks et al.^50^, Phillips et al.^25^ and Li et al.^41^. Those VOCs whose evidence was found to be more consistent and their derivatives have been highlighted.

In the last years, the urge to deeply characterize the VOCs present in BC patients’ urine has led to the need for a deeper understanding of control urine volatiles as well. Because VOCs present in urine are numerous, any analysis searching for a specific VOC will result in an extremely noisy and superposed signal with several VOC signals to be filtered out. It is long well-known that control urine consists on a mixture of water (91–96%), urea (9.3 g/dL), creatinine (0.670 g/L), sodium (1.17 g/L), potassium (0.750 g/L), chloride (1.87 g/L) and several VOCs^53^. Acetone and 2-butanone are the two predominant control VOCs in urine. Mochalski et al.^54^ performed a selective reagent ionization time of flight mass spectrometry and gas chromatography and headspace solid-phase microextraction to determine VOCs in human urine (n = 19). A total of 16 VOCs exhibiting high incidence rates were quantified in urine. Amongst them, there were ten ketones, three volatile sulfur compounds and three heterocyclic compounds (furan, 2-methylfuran, 3-methylfuran). According to this study, acetone (${\text{C}}_3{\text{H}}_6{\text{O}}$·${\text{NO}}^{+}$) has a parent ion m/z of 88.04, and when analyzed with a GC–MS, it appears at retention time Rt = 16.08 min, very early, at an intensity of 3.0–52,000 nmol/L, i.e. extremely variable among samples. 2-Butanone ($C_4H_8O$.$NO^{+}$) has a parent ion m/z of 102.06, and when analyzed with a GC–MS, it appears at retention time Rt = 22.22 min, at an intensity of 0.9–637 nmol/l, i.e. its peak is less intense than acetone’s. Figure 7 summarizes the main findings of VOCs in urine according to most authors. Further investigation in this direction might allow the technology under study to capture a less noisy signal and therefore achieve a better classification.

### Further directions

The results presented in this paper indicate that a bigger sample size would need to be analyzed and fed to a ML algorithm before one can assume that an algorithm can undoubtedly discriminate between breast cancer and control samples. Our observations indicate that the (a) urine contains enough information to allow discrimination between early-stage breast cancer and control class; and (b) if our technology was further improved, we might be able to capture more of the information contained in the sample. In conclusion, the next directions of this project include further training our AI model with a greater sample size, designing new sensors to acquire more information from the sample, and building a more robust system that performs with higher repeatability and robustness. The expected outcome upon completion of these future steps is a new version of the device that performs with higher accuracy and that can potentially be applied to breast cancer screening in the future.

## Methods

### Overall system design

System design consisted of three steps: identifying the targeted VOC biomarkers; collect urine samples to gather data; and building the final prototype based on previous observations.

#### 1st prototype: proof of concept

**Figure 8:**
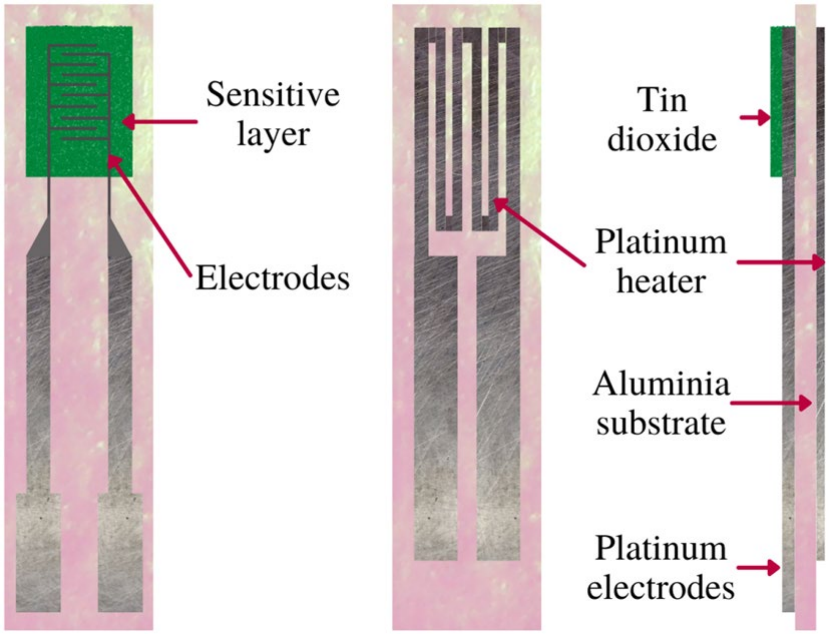
Structure of a metal oxide sensor. The sensitive layer on the top reacts with the presence of certain VOCs and thus sensors change in conductivity. The platinum heater placed on the bottom of the sensor consists of a dissipating resistance that outputs heat. By heating the sensor a higher VOC specificity is reached.

The proposed eNose is based on a metal oxide sensor array that scans the olfactory imprinting of the initial part of the urine stream. Metal oxide semiconductors are widely used when designing an eNose since they are easily available on the market, non-expensive, and notably well-performing. As depicted in Fig. 8, their surface conductivity changes upon adsorption and subsequent reaction of gases with the already-adsorbed oxygen. Since this reaction is an oxidation or reduction one—in the case of SnO or ZnO sensors-, the concentration of electrons available for conduction gets altered and causes this material to be an optimal semiconductor^45^.

**Figure 9:**
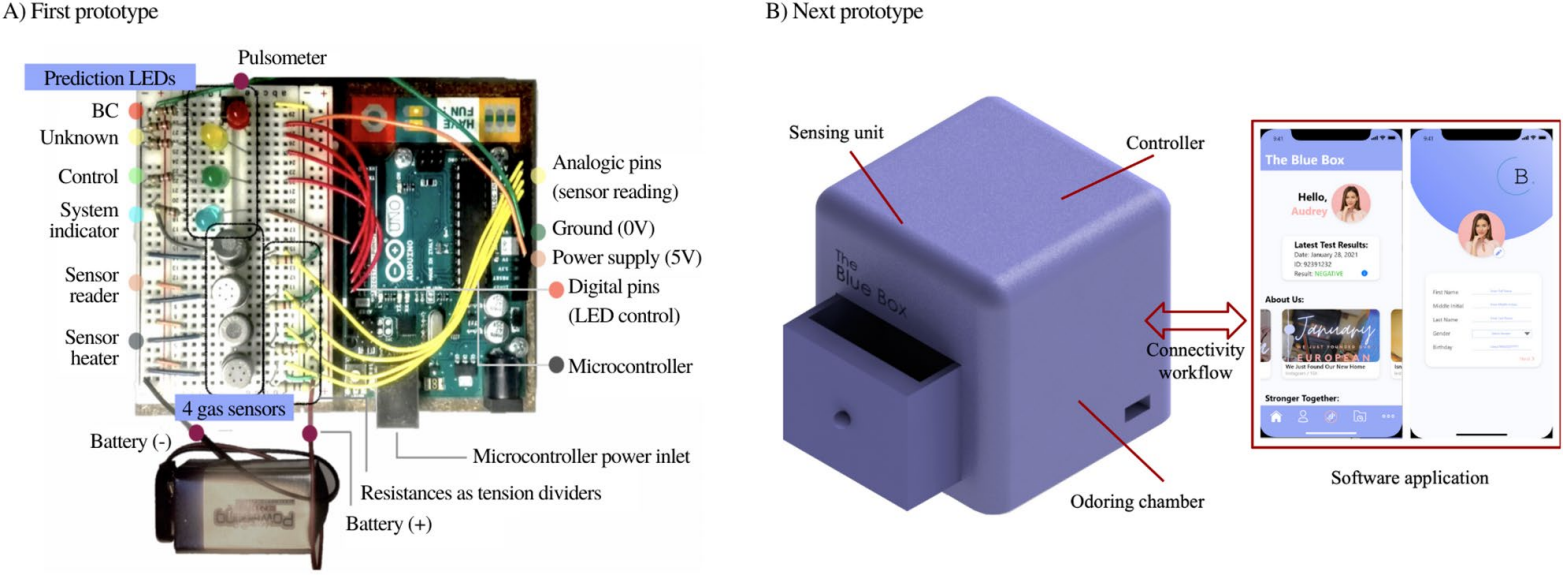
(**A**) First prototype: Main electronic components that integrate the first eNose prototype. The 4 gas sensors sniff the sample and send a VOC-dependent 0–5 V signal to the Arduinos analogic pins. Their combined signal is then reduced in dimensionality and undergoes sample classification. Depending on the prediction, output digital pins supply current to the corresponding LED, alerting of “possibly BC” “prediction no strong enough”(Blank) or “possibly control”(CTR). (**B**) Final prototype: Structure of the device and software application that we mentioned above. The 3D-printed structure contains all parts involved in the urine analysis. The software application is the interface to trigger the analysis and receive the results.

This proof of concept was built to prove whether the analysis of human urine could be performed in a portable and low-cost way without significantly decreasing the amount of information contained in the captured *smellprint*. It consisted of a breadboard with 4 VOC-specific sensors able to capture the *smellprint* of urine as well as a microprocessor to control the system. Data was analyzed on edge, and results were displayed through a color-coded LED-based user interface shown in Fig. 9A. The four gas sensors on the breadboard are the key components of the electronic system thanks to the fact that the ${\text{SnO}}_2$ layer (Fig. 8) absorbs the VOCs present in the sample and thus changes in conductivity^45^. However, this phenomenon is only possible at a given temperature, which is why 5 V need to be continuously supplied to the sensor’s heater plate, which keeps the sensitive layer warm. The heater is an $83\,{\Omega }$ resistance working at 42 mA. Finally, metal oxide sensors are poorly selective in ambient temperature with a high presence of water vapor^55^, and thus reach optimal performance at 68F, 65% HR. Arduino board also outputs voltage and ground to feed the electronic components of the system. In this case, the 5 V and GND outputs were used (Fig. 9A, orange and green wires). This is the prototype that was used to acquire the 44 urine *smellprints* mentioned in the “Results” section.

#### eNose final prototype

Based on the proof of concept mentioned above, this second prototype was aimed at better capturing the signal (i.e., to profile the smell of the sample more accurately) and allowing for a more complex algorithm: The new prototype’s connectivity workflow makes it possible to host the algorithm on the cloud, allowing it to use more computational power, i.e., being more complex. This prototype, shown in Fig. 9B, was not tested in a clinical setting. It consists of a 3D-printed structure that contains the following:

- *Odoring chamber* Physical structure where to collect the smell of urine. The chamber consists of an irradiating wall that dissipates heat over time in a controlled fashion. By doing so, it is easier to assess which VOC is evaporated at every instant of time, depending on their boiling points.
- *Sensing unit* Array of sensors that acquire the *smellprint*.
- *Controller* Microprocessor that gathers all data from the sensors and executes the embedded code.
- *Connectivity workflow* Antenna and microchip that send the captured information via Bluetooth Low Energy to a software application in a smartphone, which will, in turn, send it via the internet to the cloud-based server.
- *Cool & clean system* Set of 2 fans and air conduits through the 3D-printed case creating an airflow through the device to keep the electronics from overheating and cleans the Sensing unit once the analysis has finished.
- *Software application* Mobile application installed on the user’s smartphone to collect her demographic data, merge it with sensor data and forward it to the cloud.
- *Cloud-based server* Remote server hosting an AI-based classification algorithm that outputs the probability of a user having BC.

**Figure 10:**
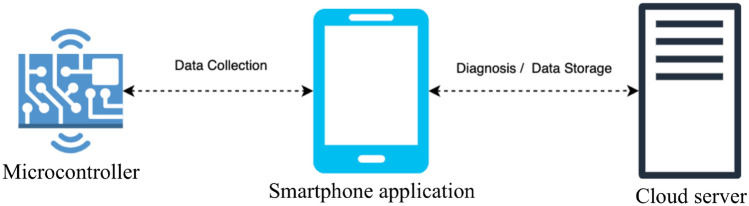
Connectivity workflow to gather data *on edge* and perform sample classification on the cloud.

As detailed in Fig. 10, the connectivity workflow of the aforementioned technology mentioned above works as follows: When a urine sample is introduced inside the device, the sensors perform a change in voltage depending on the nature of the chemical compounds present in the urine sample. Once the microprocessor has acquired the sensors’ signal, the BLE module sends the sample’s *smellprint* to the user’s phone, where the software application is installed, for 30 min. The app then sends this signal to the cloud via WiFi, where the AI-based classification AI algorithm is allocated. The algorithm classifies the sample with 95% accuracy. The signal is now not processed on edge (as in the previous prototype) but on the cloud to allow better computational power and thus better model accuracy.

### Sample collection

Urine samples were collected at the Southern Catalonia Institute of Oncology (Reus, Spain). Patient selection was carried out by a medical oncologist with experience in BC. Patients had locally advanced or metastatic BC (stages III and IV), thus presenting a high tumor burden and therefore potentially an increased concentration of excreted metabolites in urine. Control subjects were invited to participate in the study as well. These samples were obtained from a random population segment. Urine samples were stored at 4 $^\circ$C (39.2$^\circ$F) for no longer than 48 h before analysis so that 80% of the intensity of their VOCs was preserved. There is no evidence of freezing the sample harming its odor pattern^56,57^.

Rovira i Virgili Institute of Medical Research’s ethics committee approved the research. All research was performed following the provided good practice guidelines. Informed consent was obtained from all participants and/or their legal guardians. Only non-identifiable patient data was used.

Human urine is highly dependent on diet, lifestyles, ethnic background and other demographic features^42^. On the other hand, PCA works based on sample variability, taking the assumption that variability is merely based on class. To ensure this consideration does not affect the performance of the model, this assumption has been made: Human urine sample variability performs regardless of subject age. The control urine sample sub-space has a size of 51, and s0amples were collected from women aged 18–78 years (29.7 on average, with a standard deviation of 15.8). The breast cancer urine sample sub-space has a size of 39, and samples were collected from women aged 29–75 years (54.9 on average, with a standard deviation of 12.0).

Additionally, control subjects that reported being on a vegan or vegetarian diet were expected to follow a different fashion when it comes to sample prediction because a vegan diet can result in a decrease of inflammatory processes in the body and thus alters its physiology. A urine sample was taken from a 19-year-old control woman who had previously overcome thyroid cancer. In the PCA scoreplot, this sample lies in the vicinity of the BC-CTR threshold, which falls within the expected area because women who have previously overcome cancer and are currently healthy might present some cancer-like alterations in urine as well^25^. This sample has not been used for model training as specified in the patient inclusion requirements. Similar to that, one participant from the breast cancer patient group who was subject to a ketogenic diet also lies near the boundaries between cancer and control. This might be because a ketogenic diet causes pathogenic cells to become more sensitive to adjuvant cancer treatments^58^.

### Experimental validation

The following samples were obtained from recruited subjects:

- 49 urine samples from *control subjects*.
- 37 urine samples from *BC patients*.

#### Urine analysis using GC–MS

As shown in Fig. 2, urine samples have been firstly experimentally tested using a gas chromatography-mass spectroscopy. This procedure aimed at determining whether the GC–MS was sensible enough to discriminate BC samples from control samples—thus obtaining evidence that there existed enough difference between the two classes. Hence, the first step of sample classification was performed with GC–MS data.

The following protocol has been applied to the 90 human urine samples: We used a Focus DSQ II GC–MS and Triplus autosampler by Thermofisher Scientific (Fabrication number 12550090). Xcalibur and DSQ Tune II softwares were used to identify VOCs within the output signal and to visualize it. VOCs circulated along a 30 m $\times$ 0.25 mm 0.26 ţm HP-5MS column. Helium was used as the carrier gas (1.5 mL/min flow).

GC–MS vacuum setup and machine calibration have been performed according to the following parameters: Vacuum has been applied for 24 h before calibration. MS injector temperature was 220 $^\circ$C; MS transfer line temperature was 200 $^\circ$C; MS ion source temperature was 250 $^\circ$C; MS column flow was 1.5 mL He/min; Acquisition rate was 20 Hz; GC oven temperature was 200 $^\circ$C; GC Internal ambient temperature was 23 $^\circ$C; GC RF generator temperature was 28 $^\circ$C; GC slope was set to stay at 70 $^\circ$C for 0.2 min, heat up at + 10 $^\circ$C/min up to 270 $^\circ$C, and stay at 270 $^\circ$C for 10 min. Once calibrated, sample analysis has been performed according to the protocol that follows.

1. Aliquot a sample in three 11-mL vials for triple analysis.
2. Heat the sample in the oven at 150 $^\circ$C for 20 min.
3. Inject the micro-syringe through the vial silicon septum and extract 6 ţL headspace gas.
4. Quickly introduce the 6 ţL of the sample headspace into the GC–MS inlet.
5. After 30.20 min the analysis will have finished.
6. Analyse a blank vial (filled with distilled water alone) for every 4–5 samples.

## Data Availability

The data that support the findings of this study are available from the corresponding author, J.G.B., upon reasonable request.
